# Effect of Ga on the Oxide Film Structure and Oxidation Resistance of Sn–Bi–Zn Alloys as Heat Transfer Fluids

**DOI:** 10.3390/ma13235461

**Published:** 2020-11-30

**Authors:** Qingmeng Wang, Xiaomin Cheng, Zhi Liu, Zean Lv, Qianju Cheng

**Affiliations:** 1 School of Mechanical and Electrical Engieering, Huanggang Normal University, Huangang 438000, China; wangqingmeng@whut.edu.cn (Q.W.); lz3839@126.com (Z.L.); lvzean@163.com (Z.L.); qianju1990@163.com (Q.C.); 2 Hubei Zhongke Research Institute of Industrial Technology, Huangang 438000, China; 3 School of Materials Science and Engineering, Wuhan University of Technology, Wuhan 430070, China

**Keywords:** oxide film structure, oxidation kinetics, Sn–Bi–Zn–Ga, oxidation resistance

## Abstract

The effect of gallium on the oxide film structure and overall oxidation resistance of low melting point Sn–Bi–Zn alloys was investigated under air atmosphere using thermogravimetric analyses. The liquid alloys studied had a Ga content of 1–7 wt.%. The results showed that the growth rates of the surface scale formed on the Sn–Bi–Zn–Ga alloys conformed to the parabolic law. The oxidation resistance of Sn–Bi–Zn alloys was improved by Ga addition and the activation energies increased from 12.05 kJ∙mol^−1^ to 22.20 kJ∙mol^−1^. The structure and elemental distribution of the oxide film surface and cross-section were found to become more complicated and denser with Ga addition. Further, the results of X-ray photoelectron spectroscopy and X-ray diffraction show that Ga elements accumulate on the surface of the liquid metal to form oxides, which significantly slowed the oxidation of the surface of the liquid alloy.

## 1. Introduction

Fusible alloys are widely used in solar power generation, chip-heat dissipation, and nuclear power systems due to their excellent thermal properties and high-temperature stability [1,2,3]. Among the various low-melting-point alloy systems, tin-based alloys have been extensively studied [4,5]. As studied previously, fusible metals can be used as heat transfer medium in solar energy fields [6,7]. The thermal stability of alloys and their encapsulating materials is the key factor of heat transfer systems in the long-term service process at high temperature. In the actual test, there are still serious oxidation phenomena during the preparation process and high-temperature use, which greatly affects the service life of the heat transfer fluid. In addition, oxidation resistance is the key to high-temperature performance of alloy, especially in an air environment [8,9,10].

The oxidation resistance of a given alloy material depends on its ability to form oxide film on the surface. It is well known that the oxide film is formed in the oxidation process of the alloys, but their oxidation behavior varies greatly due to the differences in elemental content and preparation process. There are several publications concerning the oxidation behavior of alloy materials [11,12]. Zhao et al. [13] found that the addition of 2.5 at.% Al to Cu–20Ni–Cr alloy contributed to forming continuous external scales of chromia under lower Cr content. In Wang et al.’s study [14], the issuerable Al segregation to the surface led to the formation of Al_2_O_3_ film, which improved the oxidation resistance of the alloy. The added Nb element (1–2 wt.%) made the scale structure of the Ni–25Cr–15Fe alloys change from multiple layers to a single layer of Cr_2_O_3_, and also had a positive effect on oxidation kinetics [15]. In terms of coating surface technology, Wail Al Zoubi et al. [16,17,18] found that the use of controlled crosslinking agents for interfacial reactions on porous inorganic surfaces has better corrosion protection, light corrosion stability and antibacterial properties, and 8–HQ flower-like structure is likely to have important applications in biosensors, antioxidants, biological analysis devices, and industrial catalysis.

However, after developing new low melting point alloy heat transfer materials and studying their thermal properties and micromorphology [19], there have been seldom reports on high-temperature oxidation of liquid fusible alloys, let alone completed researches on the surface evolution of the alloys. Therefore, this work studied the oxidation behavior of Sn–Bi–Zn–Ga alloys at high temperature to reveal their oxidation mechanism, and further determined the effect of Ga addition on the improvement of the oxidation resistance of Sn–Bi–Zn alloys.

## 2. Materials and Methods

Appropriate proportions of pure Sn, Bi, Zn and Ga metals were placed in a temperature-controlled electric resistance furnace at 350 °C. Argon gas was used in the melting process to prevent the surface of the liquid alloys from being oxidized, meanwhile, the mixtures were stirred continuously to prepare uniform Sn–Bi–Zn–xGa (x = 1–7 wt.%) alloys, which were then cast into a desired shape. Component analysis was performed using X–ray fluorescence (XRF, Axios advanced). The alloys with different compositions prepared in the experiment are shown in Table 1.

The surface oxidation of the alloys was tested using a thermogravimetric (TGA, STA449F3, NETZSCH, Selb, Germany). Further, 25 mg alloy powders were placed on corundum crucibles to ensure that the alloy pellets remained substantially spherical during the experiment, which was carried out in air atmosphere with a heating rate of 10 °C·min^−1^. The oxidation rate of the alloys was expressed by the mass change per unit surface area, and the surface area of each alloy pellet was calculated by dividing the mass by the density. The density deviation of the alloys between liquid and solid state was neglected in the calculation. That is to say, it was believed that the density of the alloy sphere was constant during the heating process.

Structure observation and micro-area composition analysis of the free oxidation surface of the samples were performed using scanning electron microscope (SEM, JSM–IT300, JEOL Ltd., Akishima, Tokyo, Japan) and energy spectrum system (EDS, INCAX–ACT, Oxford Instruments, Abingdon, UK). The composition of the oxide surface was investigated by X-ray photoelectron spectroscopy (XPS, ESCALAB 250Xi, ThermoFisherScientific, Waltham, MA, USA) and X-ray diffraction (XRD, D8 ADVANCE, Bruker, Karlsruhe, Baden-Württemberg, Germany). The samples were cubes of 5 × 5 × 5 mm, which were oxidized at 400 °C for 4 h. In order to form a desired oxide layer on the surface of the alloy, oxidized slag should be avoided during the oxidation.

## 3. Results and Discussion 

### 3.1. Oxidation Kinetics 

**Figure 1:**
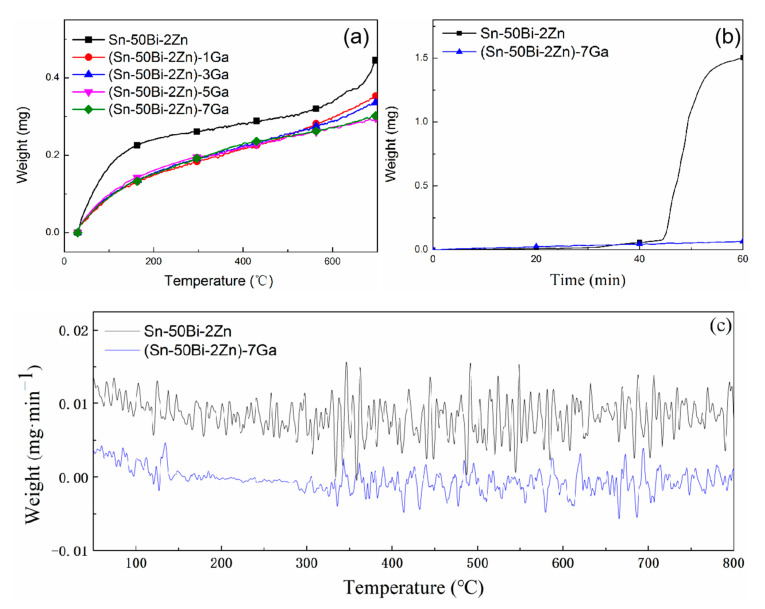
TG curves of the test alloys. (**a**) Weight gain curves with temperature, (**b**) weight gain curves with time, and (**c**) weight gain per minute with temperature.

Figure 1a shows the changing trend of oxidation weight change of sample alloys with different Ga element with increasing temperature from 0 to 700 °C. The oxidative weight gain of the four alloys with Ga was significantly lower than that of the Sn–50Bi–2Zn, indicating that the addition of Ga improved the oxidation resistance of the alloys. The weight increase of Sn–Bi–Zn alloy without the addition of Ga was large, and the oxidation weight gain when heating to 700 °C was 0.45 mg. When adding 1–5 wt.% Ga, the oxidation weight gain was 0.35, 0.33, and 0.29 mg, respectively. The oxidative weight change of the samples in the liquid state gradually decreases. As the Ga content continued to increase to 7 wt.%, the oxidative weight gain of the alloy slowed down. The reason was that Ga segregated to the surface or subsurface of the liquid alloys and was oxidized by a displacement reaction to form a barrier layer, which inhibited further oxidation of the alloys, thereby improving the oxidation resistance of the alloys. The curves of oxidation weight gain of the samples with time at 700 °C are shown in Figure 1b. It was obvious that the oxidative weight gain of the Sn–Bi–Zn matrix increased rapidly after 40 min, because the oxide of Sn cannot be dissolved in the matrix under long-term high-temperature oxidation, and the nucleation alone grew, thereby destroying the continuous compactness of the oxide film itself and increasing the defect concentration of the oxide film, which accelerated the oxidation of the alloy surface [20,21]. The curves of oxidation weight gain per minute of the two alloys as a function of temperature are shown in Figure 1c. The oxidative weight gain of the (Sn–50Bi–2Zn)–7Ga alloys was smaller than that of the matrix alloys at the same temperature.

It can be seen from Figure 1 that the weight of the liquid alloy increases rapidly in the initial stage of oxidation. The reason for the larger oxidation rate before 200 °C in Figure 1c is that the surface of the liquid metal has not yet formed an oxide film and can be in direct contact with oxygen. Therefore, the slope of the curve in the initial stage in Figure 1a is relatively large. As the temperature increases, an oxide film on the surface of the liquid metal gradually forms. The oxidation rate in Figure 1c tends to a fixed value, and the oxidation weight gain in Figure 1a also increases linearly. According to the Fromhold model [22], oxygen molecules collide with the metal surface in the initial stage of oxidation, followed by physical adsorption of oxygen molecules, and then decomposition and chemical bonding of oxygen molecules, which mainly occur at the interface between the liquid metal surface and oxygen. After half an hour, the alloy oxidation weight gain slowed down, in line with the parabolic law, this stage is the diffusion control process. This stage is divided into the diffusion of metal elements and the diffusion of active materials in oxides and reactive gases. The vapor phase oxygen is separated from the metal substrate, and the reaction material can be further oxidized by the diffusion of the oxide film. The finally formed oxide film is divided into protective oxide film and non-protective oxide film. Rate constant, *k*_1_, for the linear stage was estimated by the following equation [13]:

(1) $$\Delta w/t=k_{1}$$

where Δ*w* is the weight increase due to oxide layer growth and *t* represents the reaction time.

Table 1 lists the *k*_1_ values of the Sn–Bi–Zn–xGa alloys at 400 °C, which is given by the slope of the linear phase in the weighting curves. The slope from Sn–50Bi–2Zn to (Sn–50Bi–2Zn)–7Ga decreases continuously and the (Sn–50Bi–2Zn)–5Ga alloys show the slowest weight change. Adding Ga element effectively improves the oxidation resistance of the solder.

The parabolic growth of weight gain in Figure 1 followed Arrhenius law [14]:

(2) ln(k)=A−Q/RT

where *k* is the reaction rate constant, *A* is the pre-factor, *R* is the ideal gas constant, and *Q* is the activation energy of the reaction. Table 2 show the values of *Q* for Sn–Bi–Zn–xGa alloys was 12.05, 15.69, 21.27, 22.45 and 22.20 kJ∙mol^−^^1^, respectively.

### 3.2. Surface and Cross-Sectional Morphology of Oxide Scale

**Figure 2:**
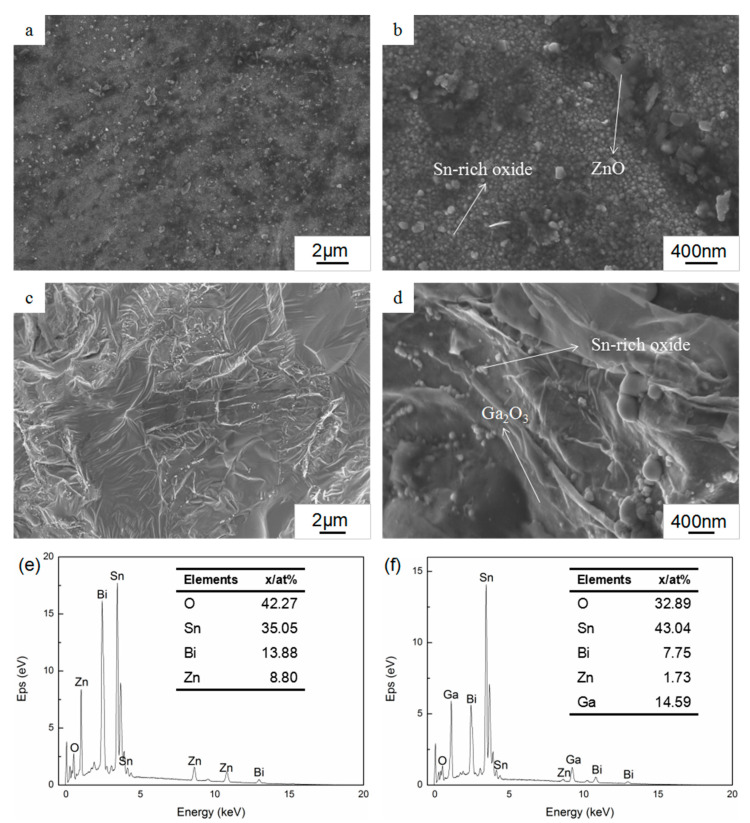
SEM images of surface morphologies of oxidation films of (**a**,**b**) Sn–50Bi–2Zn, (**c**,**d**) (Sn–50Bi–2Zn)–7Ga, and (**e**,**f**) EDS analysis of the oxide film.

Scanning electron microscopy analysis of Sn–Bi–Zn–Ga samples after oxidation for 12 h at 400 °C was performed as shown in Figure 2. The results showed that the morphology of the surface oxide layers of the two alloys was quite different. It can be seen from Figure 2a,b that there were many convex particles of different sizes distributed on the oxidation surface of Sn–50Bi–2Zn alloys, and the largest size was about 1 μm. The oxide film of Sn–50Bi–2Zn consisted of a large amount of Sn-rich oxide and a part of ZnO. The oxidized surface of (Sn–50Bi–2Zn)–7Ga in Figure 2c,d formed a relatively dense oxide film, which was much smoother and denser than that of the matrix alloys. The oxide film of (Sn–50Bi–2Zn)–7Ga was mainly composed of a large amount of Ga_2_O_3_ and a part of Sn-rich oxide. Therefore, the addition of the Ga element greatly improved the oxide film structure of the Sn–50Bi–2Zn alloys.

The results of the energy spectrum analysis of the oxidized surface are shown in Figure 2e,f. The results showed that the surface oxygen content of Sn–50Bi–2Zn alloys was higher, while that of (Sn–50Bi–2Zn)–7Ga alloys was lower, and the Ga element in the surface oxide film was much higher than the nominal content of Ga in the alloys. This indicated that the Ga element segregated to the surface of the liquid alloys during oxidation to form a dense oxide film, which hindered further oxidation of the surface of the liquid alloys. In addition, the content of Bi and Zn elements on the oxidized surface of the alloys with Ga addition decreased to 7.75% and 1.73%, respectively, indicating that the added alloying elements changed the oxidation resistance of the alloys by affecting the elemental distribution on the alloy surface.

**Figure 3:**
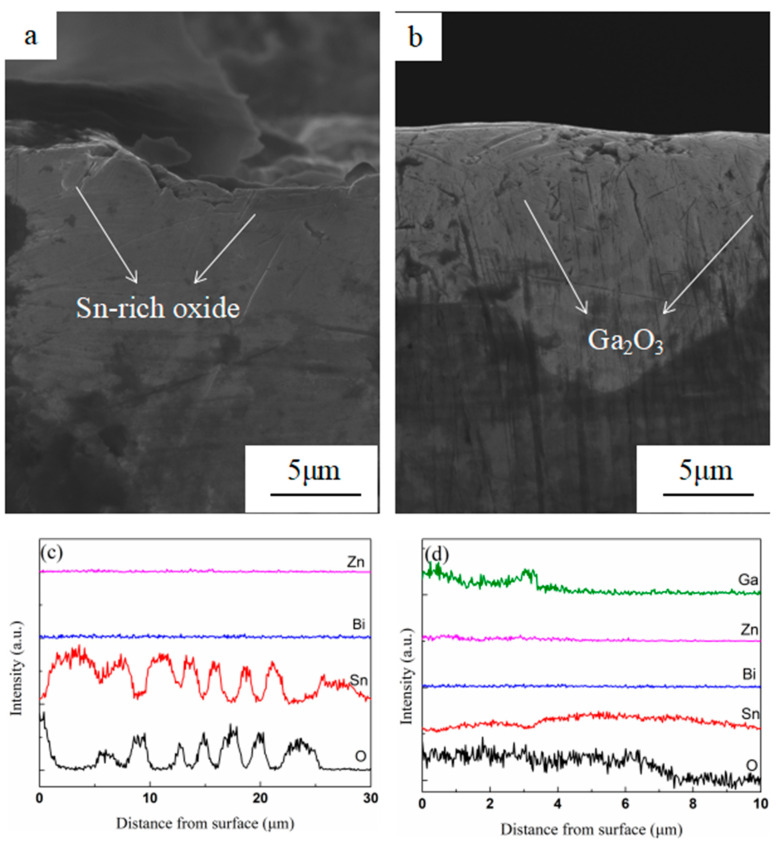
SEM image of cross section of low melting point alloys oxide film and corresponding concentration distribution of elements (**a**,**c**) Sn–Bi–Zn, (**b**,**d**) (Sn–50Bi–2Zn)–7Ga.

The cross-sectional morphology of the sample alloys after oxidation for 12 h at 400 °C is shown in Figure 3a,b, and their elemental distribution is shown in Figure 3c,d. As can be seen from Figure 3a,c, Sn-rich oxide film with a thickness of 30 μm, which was loose and porous, was formed on the surface of the liquid alloys. It was remarkable that no oxides of Bi and Zn were detected in the cross-section. In Figure 3b,d, the thickness of the (Sn–50Bi–2Zn)–7Ga surface oxide film reduced to 10 μm, and the film can be divided into two layers. The outer layer with a thickness of 4 μm was the mixed oxides of Sn-rich oxide, Bi_2_O_3_, and Ga_2_O_3_. This was because the outer oxide film was peeled off in a plurality of regions, while the element Ga in the matrix segregated to the liquid surface to form dense Ga_2_O_3_.

### 3.3. Composition of Oxide Scale

**Figure 4:**
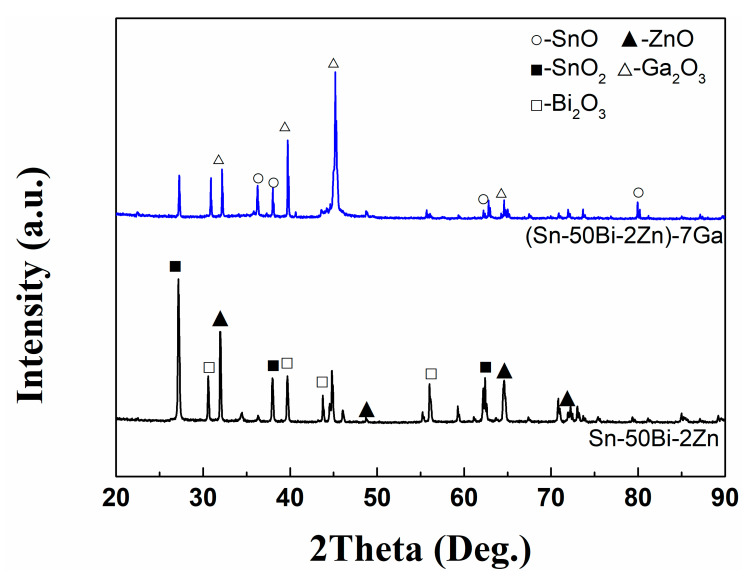
XRD patterns of oxide surfaces on the test alloys.

Figure 4 shows the XRD curve of the oxidized surface of the sample alloy. The composition of the oxide film on the surface of the two alloys and the relative content of each constituent phase were changed. The oxide film of Sn–50Bi–2Zn was composed of SnO_2_, ZnO, and Bi_2_O_3_, and the diffraction peaks of SnO_2_ and ZnO were strong. After the addition of Ga element, the oxide film was mainly composed of SnO_2_, ZnO, Ga_2_O_3_ and a small amount of SnO. It was found that the intensity of the SnO_2_ diffraction peak in the XRD pattern of (Sn–50Bi–2Zn)–7Ga was significantly weakened, and the diffraction peaks of SnO and Ga_2_O_3_ appeared. This was because the Ga element segregated to the surface of the liquid alloys to form a dense oxide film, which reduced the O content of the reaction with Sn, leading to the appearance of SnO.

**Figure 5:**
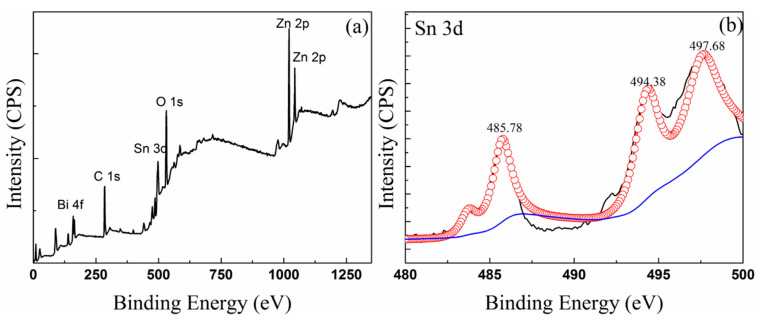

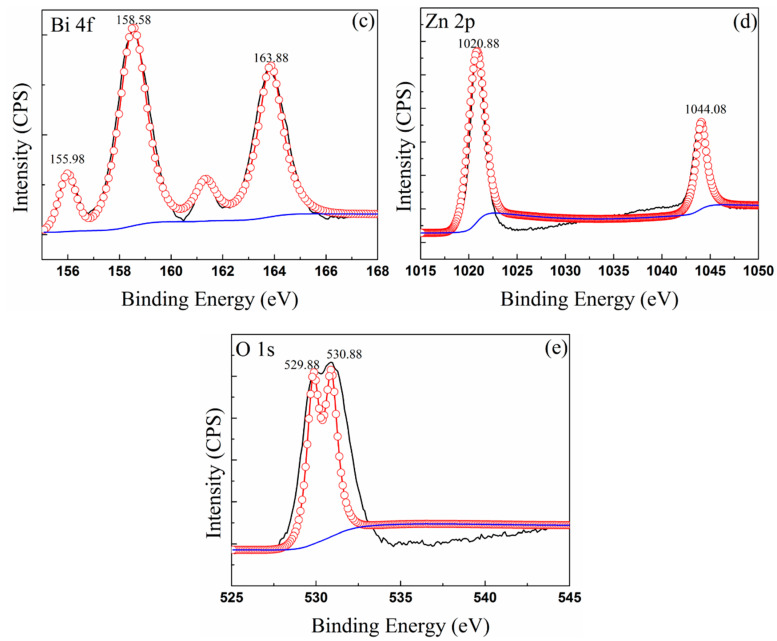
XPS spectra of the Sn–50Bi–2Zn oxide surfaces (**a**), the relevant Sn 3d (**b**), Bi 4f (**c**), Zn 2p (**d**) and O 1s (**e**).

**Figure 6:**
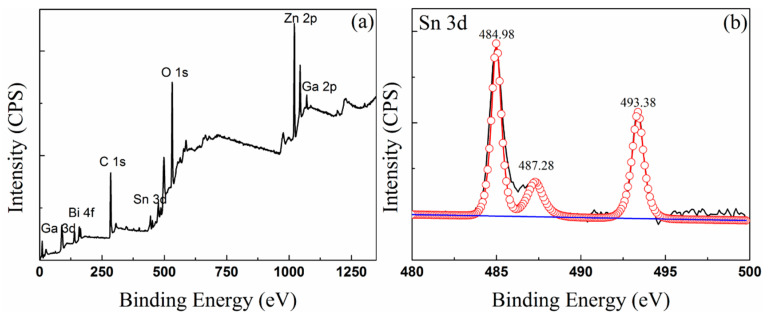

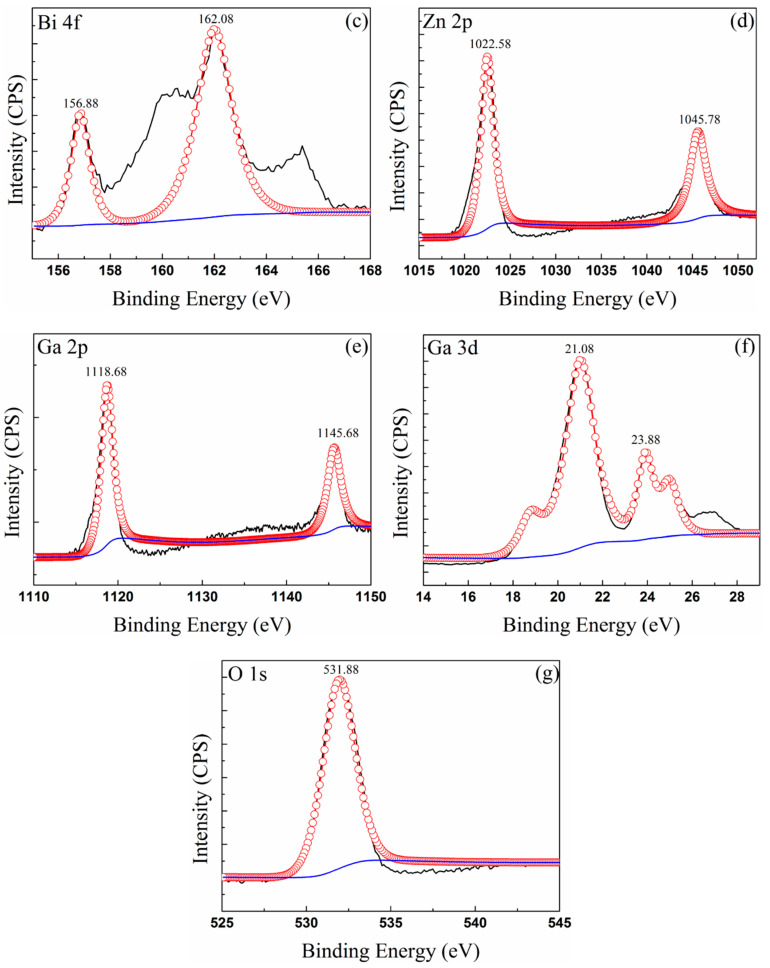
XPS spectra of the (Sn–50Bi–2Zn)–7Ga oxide surfaces (**a**), the relevant Sn (**b**), Bi (**c**), ZnO (**d**), Ga (**e**), Ga_2_O_3_ (**f**), and O 1s (**g**).

Figure 5 and Figure 6 show the XPS spectra of the oxidized surface of the Sn–50Bi–2Zn and (Sn–50Bi–2Zn)–7Ga samples, respectively, and the binding energy of related elements and oxides are listed in Table 3. Figure 5a and Figure 6a show the measured spectra of the samples with visible peaks of Sn 3d, Bi 4f, Zn 2p, Ga 2p, Ga 3d, and O 1s. Peak C1s can be considered as pollution. For more details, the selected subranges for the three Zn and Bi peaks are shown. According to the binding energy value of the XPS peak, it was found that Sn, Bi, Zn, Ga (shown only in the (Sn–50Bi–2Zn)–7Ga sample) and O coexisted in the oxidation products of Sn–50Bi–2Zn and (Sn–50Bi–2Zn)–7Ga samples. The results showed that the XPS spectrum of Zn 2p possessed two peaks corresponding to 1025.6 eV. (ZnO) and 1048.1 eV (Zn 2p1/2). It should be noted that there was no significant difference between the binding energy in the surface oxide film of the two different alloys.

The XPS spectrum of the O 1s peak is shown in Figure 5e and Figure 6g. The O 1s peak was located at 531.88 eV and may be assigned a metal oxide (OM oxygen) [23], where M may be a metal oxide formed outside the liquid alloys by Sn, Bi, Zn or Ga. The binding energy value of Ga 2p sum occurred in the range of 1110–1150 eV, and the binding energy value of Ga_2_O_3_ was 20.7 eV. These results indicate that not only the Ga oxide but also the Ga element was present on the surface of the liquid alloys, and Figure 6e,f can also prove this point.

The Bi 4f spectrum composed of a double structure (such as two peaks of Zn 2p) produced by multiple splitting is shown in Figure 5c and Figure 6c. Table 3 lists the binding energy of Bi element in different valence states. Compare the values in Table 3 and NIST database, the Bi compound on the surface of the liquid metal has two different compositions, namely, metal Bi and Bi_2_O_3_ corresponded to peak binding energy 157 and 158 eV, respectively [24]. It is important to note that the binding energy of Sn 3d shown in Figure 5e,f may be SnO, SnO_2_ or a mixture thereof [25]. SnO_2_ is the main component because of better high-temperature stability [25,26].

The results of the compositions of the oxide films measured by XPS in Table 4 show that the atomic percentages of Sn and O on the surface were higher, but the concentration of Sn was relatively lower than that of the Sn–50Bi–2Zn sample, which meant that the rate of formation of Sn oxide changed with the addition of Ga. The Sn atom concentration of the (Sn–50Bi–2Zn)–7Ga sample was reduced to about 24.14%, while the Sn–50Bi–2Zn was about 50.36%. Moreover, the atomic concentration of Ga was 33.4% on the surface, which was significantly higher than that of the matrix, indicating that Ga_2_O_3_ formed on liquid surface. The atomic concentration of Zn in Sn–50Bi–2Zn was smaller than that in the (Sn–50Bi–2Zn)–7Ga alloys, but the atomic concentration of O was large, which indicates that the surface oxide film has poor oxidation resistance.

According to the thermodynamic principle, the smaller the standard free energy of the oxide at the same temperature, the easier the metal was to oxidize. Table 5 lists the Gibbs free energy for the formation of oxides of various elements in the alloys of this study. The ability of Sn, Bi, Zn, Ga, and O to combine is Ga > Bi > Sn > Zn. It can be seen that Ga in the (Sn–50Bi–2Zn)–7Ga alloy was oxidized prior to other elements, and the oxide film on the surface of the alloy was mainly Ga_2_O_3_. The theory of high-temperature oxidation of metals suggested that the formation of a stable and dense protective oxide film by selective oxidation is one of the effective ways to improve the oxidation resistance of the alloys.

An important criterion for judging the compactness of an oxide film is the ratio of the volume of the oxide to the volume of the metal forming the oxide, PBR (Pilling−Bedworth ratio), expressed as γ [28]:

(3) $$\gamma =\frac{V_{OX}}{V_{M}}=\frac{M\rho_{M}}{nA\rho_{OX}}=\frac{M\rho_{m}}{m\rho_{OX}}>1$$

where *M* is the relative molecular mass of the metal oxides, *A* is the relative atomic mass of the metal, *n* is the number of atoms of metal per molecule of the oxide, *m* is the mass of the metal consumed to form the oxide film, *ρ*_m_ and *ρ*_ox_ are the density of the metals and metal oxides.

**Figure 7:**
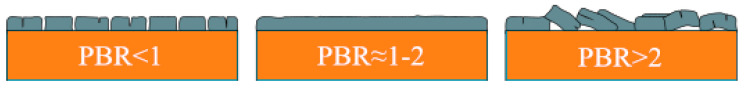
Scheme of the oxide structure and the Pilling-Bedworth ratio.

As shown in Figure 7, when PBR is less than 1, the oxide film cannot completely cover the liquid surface, which can form a complete and dense protective oxide film, and the appropriate compressive stress in the oxide film. When PBR is more than 2, the larger compressive stress in the oxide film will also crack and flake, resulting in an oxide film that does not process protective properties. Therefore, the oxide with protective properties should have a PBR value of 1 to 2 [29,30]. The calculated PBR values of ZnO and Ga_2_O_3_ are equal to 1.58 and 1.23, respectively. Accordingly, the oxide film mixed of ZnO and Ga_2_O_3_ is maintained in a stable state once formed.

## 4. Conclusions

Based on the Sn–50Bi–2Zn alloys, the addition of Ga element can change the microstructure and improve the oxidation resistance of the oxide film at 400 °C. Element Ga decreased the rate constant of the oxidation reaction and increased the activation energy. When the mass fraction of Ga in the alloys reached 5%, the Sn–50Bi–2Zn alloys processed the best oxidation resistance. The Element Ga segregated to the surface due to the small density, and preferentially reacted with oxygen to produce a dense Ga_2_O_3_ oxide film, which suppressed further oxidation of the matrix alloys, so that the oxide layer was thinned from about 30 to 10 μm. The results of SEM and XPS show that the addition of element Ga changed the structure of the oxide film on the surface of the base alloys, reduced the oxide content of Sn and Bi and formed a dense oxide film together with the oxide of Zn. This work shows that (Sn–50Bi–2Zn)–7Ga alloys can be considered as an ideal high-temperature heat transfer medium, especially when oxidation during the heat transfer process in the atmosphere is of concern.

## Figures and Tables

**Table 1:** The compositions of the alloys measured by X–ray fluorescence (XRF).

Designed Compounds	XRF Results (wt.%)			
	Sn	Bi	Zn	Ga
Sn–50Bi–2Zn	48.29	49.57	2.14	0
(Sn–50Bi–2Zn)–1Ga	47.61	49.51	1.91	0.97
(Sn–50Bi–2Zn)–3Ga	46.65	48.57	1.87	2.91
(Sn–50Bi–2Zn)–5Ga	45.62	47.58	1.86	4.94
(Sn–50Bi–2Zn)–7Ga	44.73	46.54	1.81	6.92

**Table 2:** The first stage oxidation rate constant and activation energy.

Alloys	*k*_1_ (10^−3^∙mg∙cm^−2^∙min^−1^)	*Q* (kJ∙mol^−1^)
Sn–50Bi–2Zn	7.11	12.05
(Sn–50Bi–2Zn)–1Ga	3.71	15.69
(Sn–50Bi–2Zn)–3Ga	1.37	21.27
(Sn–50Bi–2Zn)–5Ga	1.11	22.45
(Sn–50Bi–2Zn)–7Ga	1.16	22.20

**Table 3:** XPS curves of related elements and oxides in the experiment.

Elements and Oxides	Sn 3d (eV)	Bi 4f (eV)	Zn 2p (eV)	Ga 2p (eV)	Ga 3d (eV)
Sn [23]	484.9, 493.3				
SnO [23]	486.3				
SnO_2_[25]	487.3				
Bi [24]		157, 162.3			
Bi_2_O_3_ [24,25]		158.1–159.5			
Zn [26]			1021.8, 1044		
ZnO [26]			1021.7		
Ga [26]				1116.6, 1143.4	
Ga_2_O_3_ [26]					20.7

**Table 4:** The compositions of the oxide films measured by XPS (Atomic %).

Samples	Sn	Bi	Zn	Ga	O
Sn–50Bi–2Zn	50.36	5.95	9.23	—	34.47
(Sn–50Bi–2Zn)–7Ga	24.14	5.33	13.14	33.4	32.24

**Table 5:** Gibbs free energy of the elements in the experimental materials [27].

Oxidation Reaction	Δ*G*_θ_/(J·mol^−1^)			
	298 K	400 K	600 K	800 K
Sn–SnO	−302.61	−309.06	−325.03	−344.30
Sn–SnO_2_	−596.33	−602.56	−619.43	−641.61
Bi–Bi_2_O_3_	−615.89	−633.08	−675.29	−725.87
Zn–ZnO	−361.08	−366.18	−379.44	−396.05
Ga–Ga_2_O_3_	−1114.43	−1124.63	−1152.28	−1187.79
